# Autoimmunity in CD73/Ecto-5′-Nucleotidase Deficient Mice Induces Renal Injury

**DOI:** 10.1371/journal.pone.0037100

**Published:** 2012-05-29

**Authors:** Cornelia Blume, Agnieszka Felix, Nelli Shushakova, Faikah Gueler, Christine Susanne Falk, Hermann Haller, Juergen Schrader

**Affiliations:** 1 Division of Nephrology, Department of Medicine, Hannover Medical School, Hannover, Germany; 2 Institute for Transplant Immunology, Hannover Medical School, Hannover, Germany; 3 Institute for Cardiovascular Physiology, Heinrich-Heine-University Duesseldorf, Duesseldorf, Germany; Universidade de Sao Paulo, Brazil

## Abstract

Extracellular adenosine formed by 5′-ectonucleotidase (CD73) is involved in tubulo-glomerular feedback in the kidney but is also known to be an important immune modulator. Since CD73^−/−^mutant mice exhibit a vascular proinflammatory phenotype, we asked whether long term lack of CD73 causes inflammation related kidney pathologies. CD73^−/−^mice (13 weeks old) showed significantly increased low molecule proteinuria compared to C57BL6 wild type controls (4.8≥0.52 vs. 2.9±0.54 mg/24 h, p<0.03). Total proteinuria increased to 5.97±0.78 vs. 2.55±0.35 mg/24 h at 30 weeks (p<0.01) whereas creatinine clearance decreased (0.161±0.02 vs. 0.224±0.02 ml/min). We observed autoimmune inflammation in CD73^−/−^mice with glomerulitis and peritubular capillaritis, showing glomerular deposition of IgG and C3 and enhanced presence of CD11b, CD8, CD25 as well as GR-1-positive cells in the interstitium. Vascular inflammation was associated with enhanced serum levels of the cytokines IL-18 and TNF-α as well as VEGF and the chemokine MIP-2 (CXCL-2) in CD73^−/−^mice, whereas chemokines and cytokines in the kidney tissue were unaltered or reduced. In CD73^−/−^mice glomeruli, we found a reduced number of podocytes and endothelial fenestrations, increased capillaries per glomeruli, endotheliosis and enhanced tubular fibrosis. Our results show that adult CD73^−/−^mice exhibit spontaneous proteinuria and renal functional deterioration even without exogenous stress factors. We have identified an autoimmune inflammatory phenotype comprising the glomerular endothelium, leading to glomeruli inflammation and injury and to a cellular infiltrate of the renal interstitium. Thus, long term lack of CD73 reduced renal function and is associated with autoimmune inflammation.

## Introduction

While the intracellular formation of adenosine predominates under hypoxic conditions, its extracellular formation results from the degradation of extracellular adenine nucleotides by action of CD39/ecto-apyrase and CD73/ecto-5′-nucleotidase [1], [2]. The extracellular cAMP-adenosine pathway may be another important source for adenosine formation [3]. CD73 is a 70 kDa GPI-anchored cell surface enzyme catalyzing the extracellular conversion of nucleotide monophosphate esters into respective nucleosides [4]. CD73-deficient (CD73^−/−^) mice were independently generated in the last few years by three laboratories and have provided growing evidence that CD73-derived adenosine participates in numerous important biological functions, such as playing a crucial role in hypoxia-induced vascular leakage [5] and tissue protection as shown in a model of bleomycin-induced lung injury [6]. Moreover, CD73-deficiency is associated with a prothrombotic and proinflammatory phenotype of the vasculature, increased attachment of lymphocytes and monocytes to the endothelium and enhanced expression of VCAM-1 in aortic vessels linked to enhanced NFκB-activity exhibiting an autoimmune inflammation [7], [8].

In the kidney, adenosine has numerous effects including arteriolar vasoconstriction in the outer cortex and vasodilatation in the deep cortex and medulla, mediation of tubuloglomerular feedback, inhibition of renin release and influencing electrolyte transport along the proximal tubules [9]. Adenosine maintains the kidney structure and protects from ischemia [10]. Adenosine exerts its action via specific G-protein coupled receptor subtypes classified as A1, A2a, A2b and A3 [11] which are pharmacologically relevant due to availability of highly specific agonists and antagonists [12]. The distribution of adenosine receptors in the kidney is only incompletely defined due to the cellular complexity of the kidney and the low expression levels [9]. At the cellular level, CD73 is predominantly found in peritubular fibroblasts and glomerular mesangial cells [13]. CD73-derived adenosine has no impact on erythropoietin production but might play role for nephrogenesis since young CD73^−/−^mice [14] show a reduced kidney weight. CD73^−/−^mutants provided evidence that CD73-derived adenosine regulates tubuloglomerular feedback [15], [16], most likely mediated by A1 receptors [17], [18]. CD73-derived adenosine has also been shown to be renoprotective in a model of diabetic glomerulopathy, where stimulation of the A2A receptor resulted in structural improvement [19], possibly by stabilization of podocytes, blocking podocyte permeability and actin disruption [20]. A2A receptor activation was also found to be protective against renal injury in a mouse model of lupus nephritis (MRL/lpr mice) [21].

The aim of the present study was to investigate whether long term lack of CD73- derived adenosine in renal tissue may induce inflammation associated kidney pathologies. Given the general vascular proinflammatory phenotype of CD73^−/−^mice [7], [8] we explored whether the lack of extracellularly formed adenosine might cause kidney injury and translate into deterioration of kidney function in the long run.

**Figure 1:**
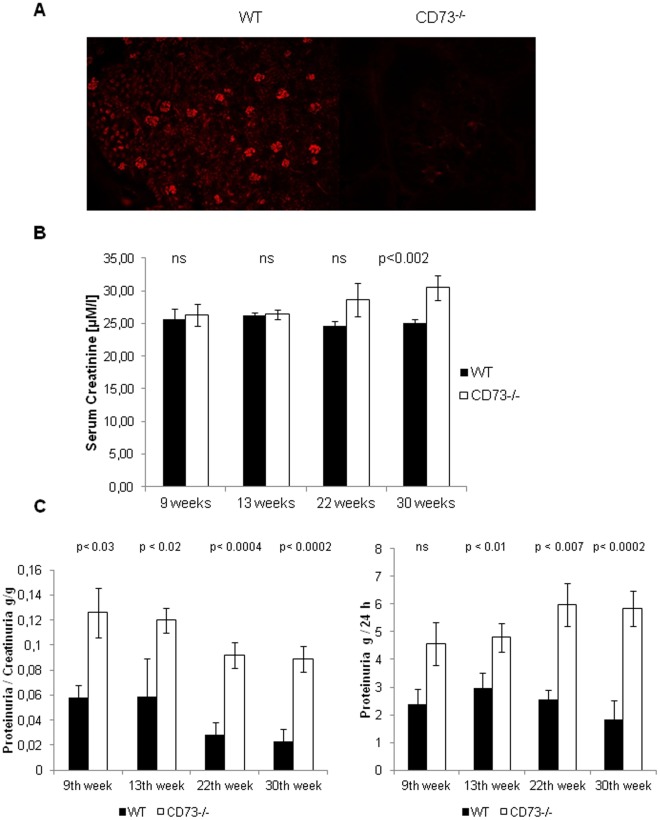
Increase of total proteinuria and decrease of renal function in mice lacking CD73. (A) Using immunohistochemistry with a CD73-specific antibody and a rhodamin red λ conjugated secondary antibody, confocal microscopy at a magnitude of 10× revealed staining of glomerular cells in the mesangium as well as peritubular cells in renal tissue of WT mice which is absent in the mutant. Images are representative of 3 of 5 animals with more than 2 sections per kidney. (B) Renal function estimated by the measurement of serum creatinine, auto normalized, in WT and CD73^−/−^mice. 8 to19 mice per group were used. Data are presented as means ± SEM, level of significance as indicated according to Student t-test. (C) Total proteinuria was elevated and increased with age in CD73^−/−^mice as compared to the wild type controls. We detected proteinuria/creatinuria in [g/g], left panel, and total urine protein in [mg/24h], right panel. 8 to 19 mice per group were used. Data are presented as means ± SEM, level of significance as indicated according to Student t-test.

## Materials and Methods

### Mouse Model

CD 73^−/−^mice were generated as previously described [7] and further backcrossed for 10 generation with C57/BL6 mice. Wild type C57/BL6 mice used for this study were supplied by Charles River Laboratories. CD 73^−/−^mice were bred under pathogen free conditions at the animal facility of the Heinrich-Heine-University (Duesseldorf, Germany) and at the Phenos GmbH (Hannover, Germany) and cared for in accordance with our institution’s guidelines for experimental animals. All experiments were approved by the animal protection committee of the local authorities: The Bezirksregierung Duesseldorf (8.87–50.10.34.08.296) as well as the niedersaechsische Landesamt fuer Verbraucherschutz und Lebensmittelsicherheit (LAVES; 33.9-42502-12-10/0031). Male mice 9 weeks to 30 weeks of age were used as indicated.

### Determination of Renal Function, Total Proteinuria and Albuminuria

From an age of 9 weeks on, mice were placed within metabolic cages every 4 weeks, and urine was collected for 24 h for determination of the ratio of total protein/creatinine in g/g and urea concentration, and urine volume was monitored. Blood samples were taken from the retrobulbar plexus for determination of creatinine, blood urea nitrogen (BUN), and creatinine and BUN clearances were calculated according to the standard formula. Albumin concentration in appropriately diluted urine samples was measured using the Albuwell M Assay Kit (Exocel, Philadelphia, PA, USA) according to the manufacturer’s instructions. At the age of 22 to 30 weeks, animals weight and kidneys weight in both mice groups (CD73^−/−^ vs. WT controls) was not significantly different.

### Gel Electrophoresis

For protein analysis of 24 h – urine samples, an “Excel Gel Homogenous” (Amersham, Bioscience, Sweden) was used loading 10 µg of total protein on each lane of the gel using a rainbow protein standard (GE healthcare, Munich, Germany). Electrophoresis was performed and a “silver staining kit” was used according to the manufacturer’s instructions.

### Preparation of Kidneys for Histological Analysis

Mice were anesthetized by intraperitoneal injection of a mixture of ketamine (60 mg/kg, Pharmacia & Upjohn, Erlangen, Germany) and xylazinhydrocholoride (10 mg/kg, NayerVital Leverkusen, Germany) and a first perfusion of the kidneys was performed with PBS via renal arteries. Thereafter one kidney was isolated and cryo-conserved in isopentane. Afterwards, a second perfusion using PBS buffered 4% paraformaldehyde (PFA, pH 7.4) was performed before the second kidney was isolated and placed in fixation solution (4% paraformaldehyde in PBS) at 4°C for further procedures.

### Histological Analysis of the Kidneys

Kidneys were embedded into paraffin or in K4M resin (Electron Microscopy Sciences; Hatfield, UK) and further processed for “periodic acid Schiff”- staining (PAS), Hematoxilyn-Eosin staining (HE), “van Gieson’s” staining, Sirius Red staining (NovaUltra TM Sirius Red Stain Kit, according to the manufacturers’instructions) or electron microscopy, respectively.

**Figure 2:**
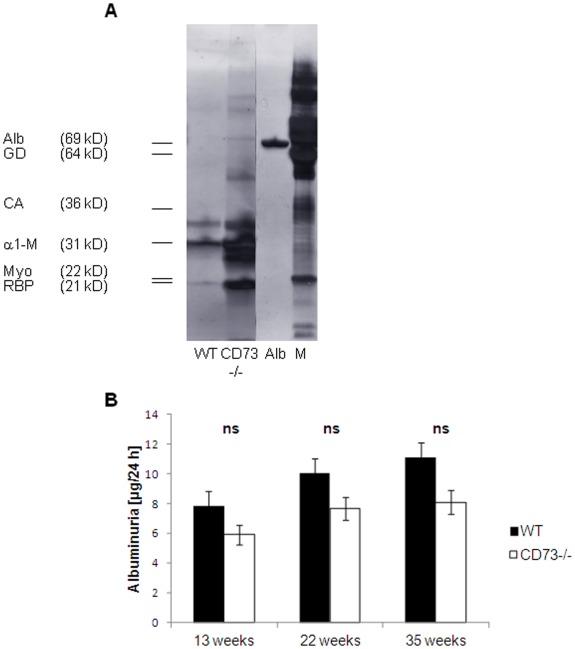
Total proteinuria in CD73^−/−^mice is a low molecule proteinuria. (A) Profiling the urinary protein content, 10 µg of total urinary protein from WT and CD73^−/−^mice were subjected per lane to a 12.5% nonreducing SDS-PAGE (silver staining). Mice groups are shown as indicated, Alb  =  bovine serum albumin, M  =  rainbowmolecule weight marker with characteristic proteins as indicated (CA  =  carbonic anhydrase, GD  =  glutamatic dehydrogenase, Myo  =  myoglobin red). Proteins bands identified by size are indicated: α1-M  =  α 1-microglobulin, RBP  =  retinol binding protein. (B) Urinary albumin excretion was not different as compared to WT mice. 9 to 24 mice per group were used. Data are presented as means SEM.

### Quantification of Collagen Deposits

Image analysis was performed by an investigator blinded to the source of the sample. Sirius red stained sections were analyzed using a polar microscope, 3–5 images of the renal cortex per animal were taken. Images were further quantified using the software “photoshop” and histogram analysis was performed for selective pixel counting with one set point for the pixel luminance used for all images. The ratio of the collagen derived - pixels versus all pixels per image were calculated.

### TUNEL-DAB Assay

For TUNEL-assay (terminal deoxynucleotidyl transferase-mediated dUTP nick-end labeling) 4 µm sections of 4% paraformaldehyde (PFA)-fixed paraffin-embedded tissues were deparaffinized, treated with the terminal deoxynucleotidyl transferase enzyme and incubated in a humidified chamber at 37°C for 1 h (S7110 Apop Tag Flourescein, Chemicon). After washing, the tissue was treated with FITC-labeled anti-digoxygenin, incubated for 1 h, and washed. Negative controls were prepared under the same conditions, with the omission of the terminal deoxynucleotidyl transferase enzyme. TUNEL positive cell numbers were counted in 20 nonoverlapping view fields each specimen without knowledge of the animal assignment.

### Quantification of Glomerular Cells

Kidneys of the CD73^−/−^ and WT animals were comparably subjected to a morphometric analysis. Semithin sections (0.5 µm) were prepared using a ultracut- microtome (Reichert, Heidelberg, Germany) and basic fuchsine and methylen blue staining were performed. Scoring was performed at a magnitude of 100× (oil immersion) using an Orthoplan microscope (Letz, Wetzlar, Germany) meeting the criteria of the semiquantitatice scoring system as proposed by El Nahas et al [22]. All semiquantitative investigations were performed in a blind manner by an observer who was unaware of the study protocol. On five semithin sections per animal, the glomerular cell number of endothelial cells, mesangial cells, capillaries and podocytes was analyzed in at least 30 glomeruli per animal using the point-counting method and a 100-point eyepiece (integration plate II, Zeiss Co.).

**Figure 3:**
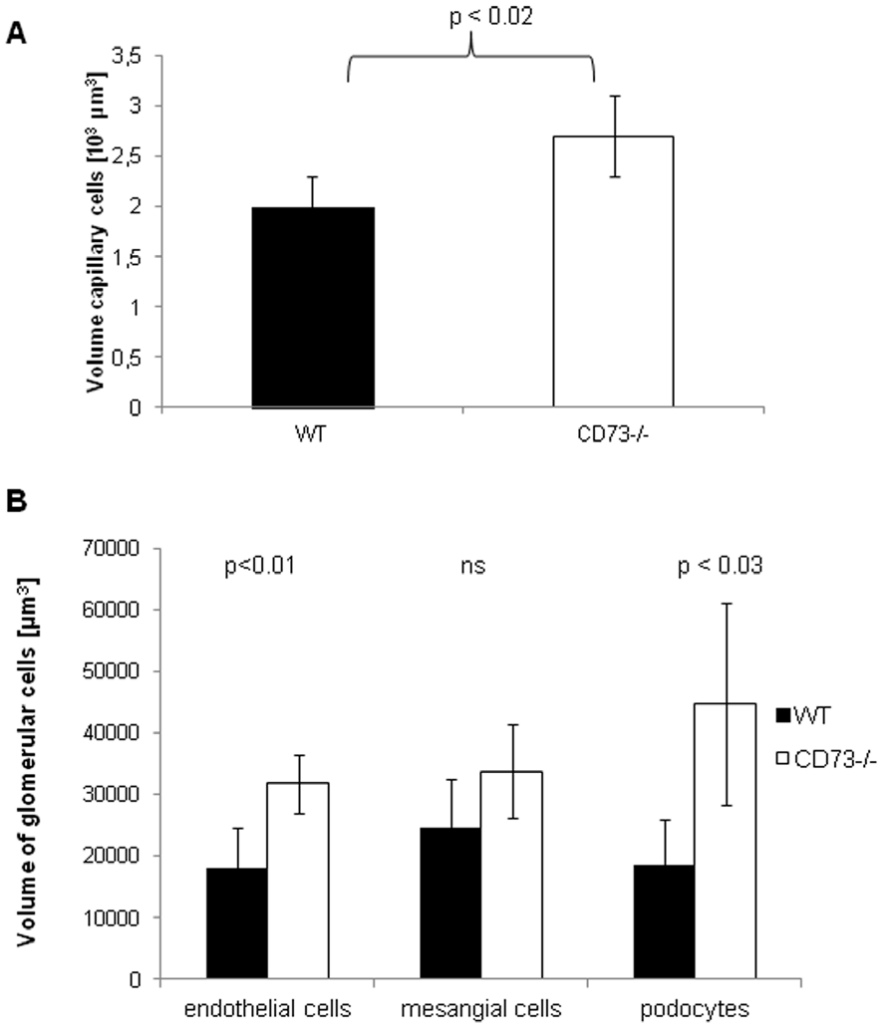
CD73^−/−^mice show an increased volume of podocytes, glomerular endothelial cells and capillaries. Five sections per animal were analyzed using the point-counting method and a 100-point-eyepiece (integration plate II, Zeiss Co.) at a magnitude of 100× (oil immersion). (A) Volume of glomerular capillaries in 10^3^ µm^3^ in CD73^−/−^mice as compared to WT (n = 5−6, means ± SD, p<0.02 according to Student t-test.). (B) Volume of glomerular cells in [µm^3^] CD73^−/−^mice as compared to WT (n = 5−6, means ± SD, p<0.01 according to Student t-test.).

### Immunohistochemistry

6 µm sections were generated from the native cryo-conserved kidneys embedded in “tissue tec” using a cryo-microtome. Tissue was fixed for 10 min with “Zamboni’s fixative” (4% PFA, 15% picrine acid in phosphate buffer, pH 7.4) and washed three times with PBS before incubation with blocking solution (10% normal goat serum or 10% donkey serum) for 1 h. The following primary antibodies were used: anti-CD73 (clone TY/23; rat, BD Pharmingen), anti-C3 (RMC11H9; rat, monoclonal; Acris Antibodies, Hiddenhausen, Germany), anti-CD8 and -CD11b (goat, polyclonal; Santa Cruz Biotechnology, CA, USA), anti-GR1 (rat anti-mouse Ly-6G and Ly-6C monoclonal, BD Pharmingen), anti-CD 25 (rabbit anti-mouse, polyclonal, Santa Cruz Biotechnology, CA, USA), anti-WT1 (rabbit anti-mouse, polyclonal, Santa Cruz Biotechnology, CA, USA). The following secondary antibodies were used as appropriate: fluorescein isothiocyanate (FITC)-conjugated anti-rat polyclonal Ab (goat, Dianova, Hamburg, Germany), rhodamine red λ conjugated anti-rat polyclonal Ab (goat, Dianova, Hamburg, Germany), Cy TM 3 labeled anti-goat Ab and anti-rabbit Ab (donkey, Dianova, Hamburg, Germany).

Paraffin sections (4 µM) were analyzed using antibodies against synaptopodin (monoclonal, Progen Biotechnik, Heidelberg, Germany; second Cy3-conjugated antibody Jackson Immuno Research Lab. Suffolk, UK) and nephrin (guinea pig anti-mouse, Progen Biotechnik, Heidelberg, Germany, second Cy3 conjugated antibody Molecular Probes, Leiden, NL). Furthermore, 6-diamidino-2-phenylindol-dihydrochloride (DAPI) was used to detect cell nuclei. Sections were examined using the Olympus System Microscope WX 60 for fluorescence microscopy and suiTable software (“Cell F”) and a Zeiss Axioplan-2 imaging microscope with the scientific image processing software AxioVision 4.6 (Zeiss, Jena, Germany).

### BioPlex Assay for Cytokines, Chemokines and Angiogenetic Factors in Serum and Renal Tissue

Snap-frozen renal tissue samples were mechanically disrupted and treated by lysis solution (Bio-Rad). After sonication, samples were centrifuged at 4500× g for 6 min at 4°C. Protein concentration in the supernatant was determined using Bradford assay and adjusted to 700 µg/ml using serum diluent (both from Bio-Rad). Amounts of chemokines, cytokines and angiogenetic factors were measured using multiplex technology (Bio-Rad) according to the manufacturer’s protocols. Serum concentrations were measured in pg/ml according to the manufacturer’s protocols. Concentrations of n = 7 mice per group were statistically analyzed using the statistical software “graph prism” and Mann-Whitney-U-Test.

**Figure 4:**
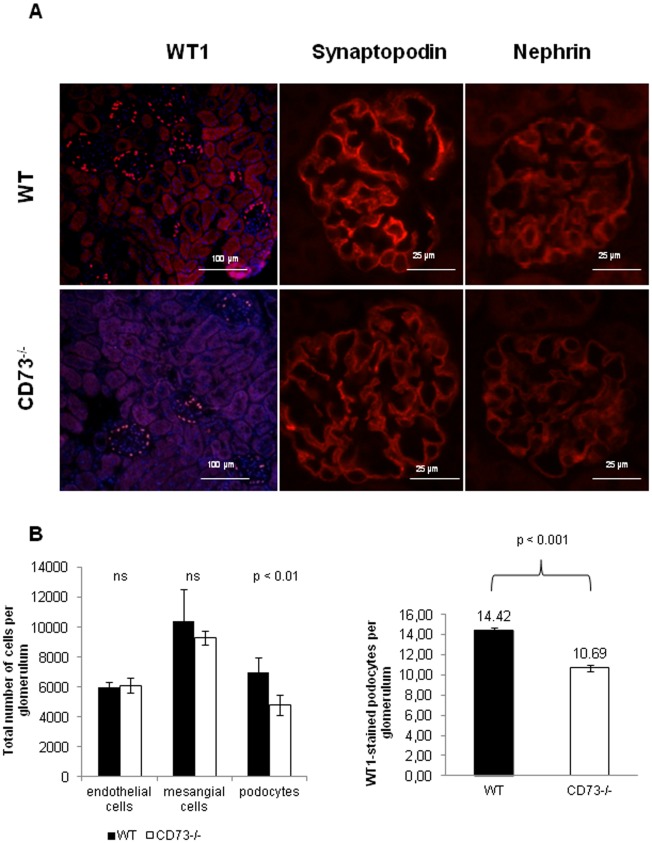
Reduced number of podcytes in CD73^−/−^mice. (A) The expression of the podocyte markers WT1, synaptopodin and nephrin was immunohistochemically detected on 6 µM cryosections of the renal cortex of CD73^−/−^mice vs. WT-mice. Images are representative of 6–7 animals per group. (B) Statistical analysis showed a decreased number of WT1-stained podocytes per glomerulus (counted in 30 glomeruli per section and 7 mice per group), means ± SEM, p<0.0001 according to Student t-test (left panel). Histomorphometrical analysis (integration plate II, Zeiss Co., magnitude of 100×, oil immersion) confirmed reduced podocytes per glomerulus as compared to other glomerular cells (n = 5−6 mice per group), means ± SD, p<0.01 according to Student t-test (right panel).

**Figure 5:**
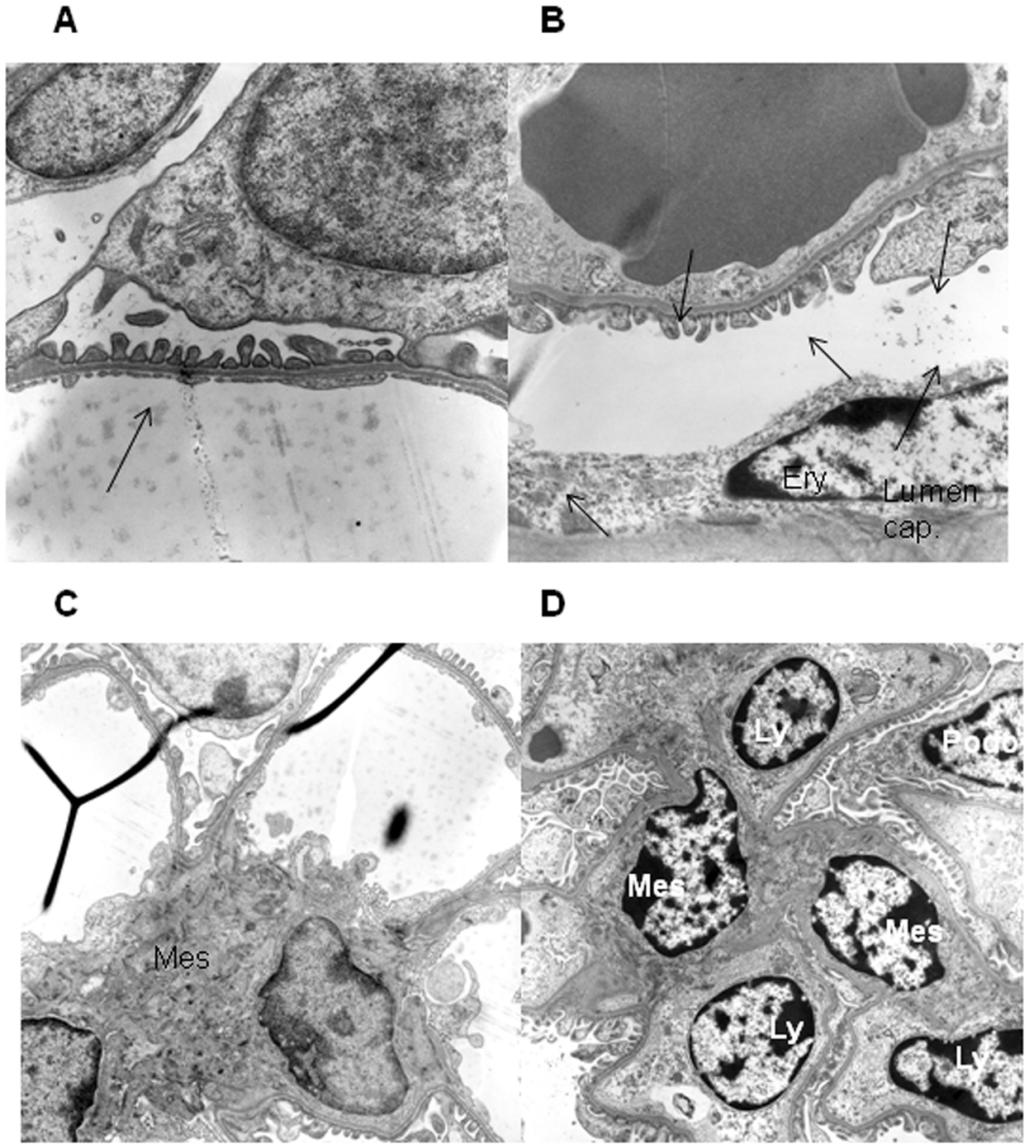
Glomerular endotheliosis and injury in CD73^−/−^mice. Electrone microscopic analysis was performed n = 7 CD73^−/−^mice vs. WT, original magnification x 10000 or 5000. (A) Ultrastructural analysis of the glomerular filtration barrier from a 3 months old WT mouse with regular appearance of foot processes and slit membranes and fenestration of the endothelium. (B) Analysis of the glomerular filtration barrier from a 3 months old CD73^−/−^ mouse shows cytoplasmic swelling apparent in endothelial cells (cap. Lumen  =  capillary lumen, ery  =  erythrocyte), endothelial fenestrations and single foot effacements were reduced (arrow). No subepithelial depositis were detectable. (C) Overview of a glomerular segment with regular appearance and without glomerulitis (3 months old WT-mouse). (D) Overview of a glomerular segment in a 3 months old CD73^−/−^mouse (mes  =  mesangial cell, ly  =  lymphocytes) shows lymphocytic glomerulitis.

## Results

### CD73 Staining of Renal Tissue

Immunohistochemical analysis demonstrated the presence of CD73 within the glomeruli as well as peritubular in renal tissue of WT mice. As expected, this was completely absent in mutant mice (Figure 1A).

### Renal Function and Total Proteinuria in CD73^−/−^mice

Renal function was analyzed in all animals by determination of the serum creatinine level. Compared to WT controls, serum creatinine level was increased in mutant mice starting at 22 weeks of age and reaching the level of significance in 30 weeks old mice (30.5±1.9 vs. 25.1±0.5 µM/l for CD73^−/−^mice and WT-mice, respectively) (Figure 1B).

We also analyzed proteinuria in CD73^−/−^mice starting at 9 weeks of age. The proteinuria/creatininuria ratio was significantly increased in 9 weeks old male CD73^−/−^mice (p<0.03) and remained increased up to 30 weeks (Figure 1C, left panel). Compared to WT controls, male CD73^−/−^mice exhibited increased level of total protein in urine already at 9 weeks of age, and a significant increase in total proteinuria was observed at the age of 13 weeks (4.80±0.52 mg/24 h vs. 2.97±0.54 mg/24 h; p<0.03), 22 weeks (5.97±0.78 vs. 2.55±0.35 mg/24 h in WT controls; p<0.01) and 30 weeks (5.83±0.651 vs. 1.83±0.651 mg/24 h in WT controls; p<0.0002; Figure 1C, right panel).

Urinary protein profiling with SDS-PAGE revealed a low molecular type of proteinuria in CD73^−/−^mice (Figure 2A) with strong protein bands running at the molecular weight of 21–31 KDa. Determination of urinary albumin excretion (Figure 2B) revealed similar results in the mutant and in the wild type.

**Figure 6:**
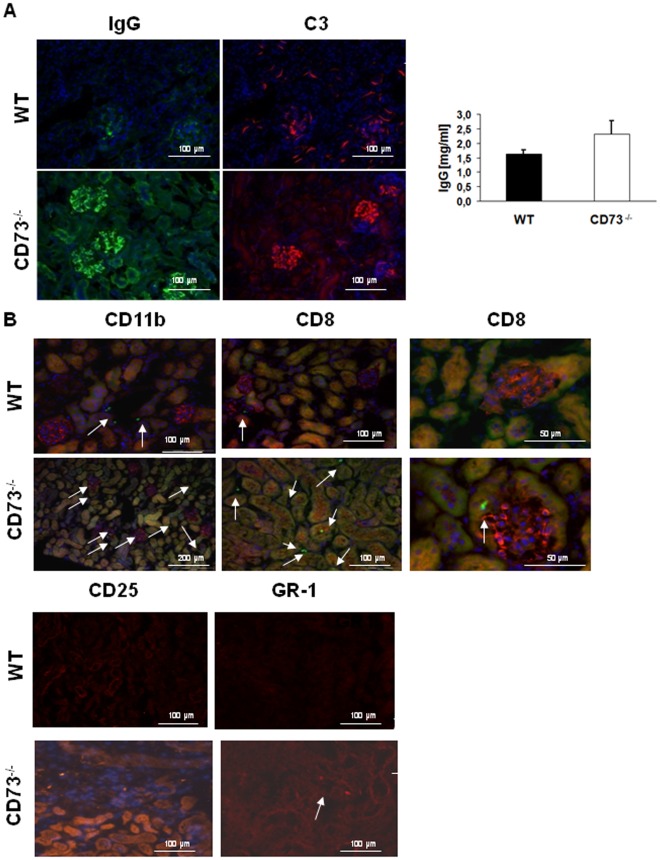
Collagen deposition in the renal cortex, peritubular and surrounding the glomeruli in CD73^−/−^mice. (A) Representative images of fibrillar collagen deposition shown by Sirius Red staining (in CD73^−/−^as compared to WT controlc mice, ×200 magnification). (B) Image analysis demonstrates that CD73^−/−^mice exhibit increased levels of fibrillary collagen deposition within the cortex compared with WT controls (significance as indicated, p<0.002 CD73^−/−^ vs WT mice, Sirius red staining, 3–5 images per animal, n = 7 animals/group, Student t-test).

**Figure 7:**
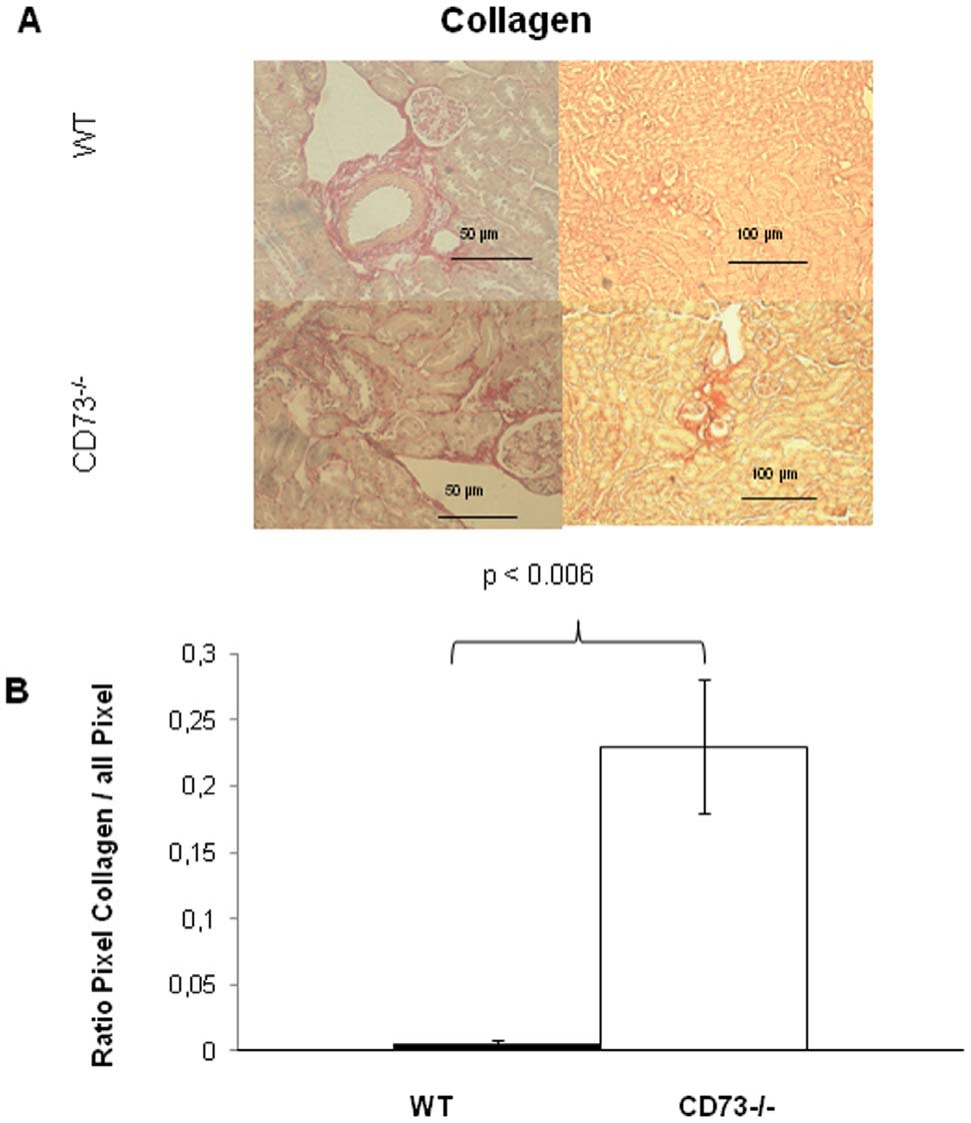
Humoral inflammation in glomeruli and cellular infiltrates in the interstitium of CD73^−/−^mice. The renal cortex of CD73^−/−^mice showed glomerular deposits of IgG and C3 as well as increased presence of CD 11b- a**n**d CD8- positive cells as well as CD25- and GR-1–positive cells in the interstitium of CD73^−/−^ mutants which were absent in WT-mice. (A) IgG staining (green, FITC conjugated secondary antibodies) and C3 staining (red, rhodamin red λ conjugated secondary antibodies) was carried out in cryo-conserved, 7-µm sections of the kidneys of WT and CD73^−/−^mice. Images are representative of >3 independent experiments. (B) CD11b and CD 8 staining (green, FITC conjugated secondary antibodies) and CD25 and GR-1 staining (red, Cy 3) was performed in cryo-conserved, 6–7-µm sections of the mice kidneys. Images are representative of 5 to 7 animals with more than 2 sections per kidney.

### Decreased Number of Podocytes but Increased Volume of Capillaries and Endothelial and Mesangial Cell Swelling in CD73^−/−^Mice

The scoring of 0.5-µm sections of kidneys in WT and CD73^−/−^mice yielded comparable results as to the number of glomeruli per kidney and for the medulla/cortex proportion within the kidneys (data not shown). Using the histomorphometric analysis described by El Nahas et al [22] the volume of capillaries was significantly increased in the mutants (2.7±0.3 10^3^ µm^3^ vs. 2.0±0.4 10^3^ µm^3^, p<0.02, Figure 3 A) In contrast, the number of endothelial cells and mesangial cells per glomerulus was comparable between the groups, but the mean volume of endothelial cells and podocytes was approx. 2-fold higher in CD73^−/−^mice (p<0.01, Figure 3B).

The reduced number of podocytes in glomeruli of CD73^−/−^mice could be also demonstrated by immunohistological analysis of the of the Wilms tumor 1-protein (WT1) which is a nuclear marker of podocytes. WT1 was down regulated in glomeruli of CD73^−/−^mice (Figure 4A), corresponding well to the histomorphometric finding of reduced podocytes per glomerulus. Additionally we saw a decrease of the synaptopodin and nephrin staining in the mutant mice vs. control mice (Figure 4 A). Figure 4B shows that the score of WT1 stained podocytes per glomerulus in CD73^−/−^mice vs. WT controls (p<0.001, right panel). Using histomorphometry, the number of podocytes per glomerulus was also markedly decreased compared to WT mice (4771.9±679.6 vs. 7005.0±966.8, p<0.01, left panel).

### Inflamed Endothelium in Kidneys of CD73^−/−^Mice

Transmission electron microscopy analysis of kidney tissue (Figure 5) revealed a swelling of endothelial cells (arrows, Figure 5B vs. A) in CD73^−/−^mice as a marker for inflamed endothelium going along with reduced fenestration. The glomerular capillaries were infiltrated by lymphocytes indicating a lymphocytic glomerulitis (Figure 5D vs. 5C), but no subepithelial deposits. In addition, we found single foot process effacements in the glomerular filtration barrier in 3/10 mutant mice which were absent in wild types (Figure 5B).

### Inflammatory Cells and C3- and IgG-deposits in the Renal Cortex of CD73^−/−^Mice

Immune histological analysis of kidney tissue showed positive glomerular staining for IgG and complement factor C3 (Figure 6A) indicating that antibodies against glomerular proteins are able to activate complement, and toal IgG level were elevated in the serum. At the cellular level, an enhanced invasion of CD11b-positive cells, presumably monocytes/macrophages into the renal cortex of CD73^−/−^ mice (Figure 6B, glomeruli and tubulointerstitium) was observed compared to WT mice. This increase in CD11b-staining was accompanied by enhanced detection of GR1-positive cells in the glomeruli suggesting that GR1^+^CD11b^+^ proinflammatory monocytes are recruited in the absence of CD73. In addition, elevated infiltration of CD8-positive cells, presumably cytotoxic T cells (CTL) was detectable in CD73-deficient mice (Figure 6B). These CD8^+^ T cells seemed to be activated since an enhancement of CD25-positive cells next to the vasculature in the interstitium of CD73^−/−^mice (Figure 6C) was also observed that was absent in WT control mice. In contrast CD4-positive cells were not found in the renal tissue, neither in CD73^−/−^nor in WT mice (data not shown) thus, excluding that the CD25^+^cells represent CD4^+^CD25^+^ regulatory T cells. Using sirius red staining and image quantification as mentioned above, we found enhanced collagen deposition in the renal cortex of older CD73^−/−^mice vs. WT controls of the same age (30 weeks, p<0.006, Figure 7 B). Collagen was found peritubular and surrounding the glomeruli, but not within the glomeruli (Figure 7 A).

### Upregulation of IL-18, VEGF, TNF-α and MIP-2 (CXCL-2) in Serum of CD73^−/−^ Mice, Unaltered Chemokines and Cytokines in Renal Tissue

**Figure 8:**
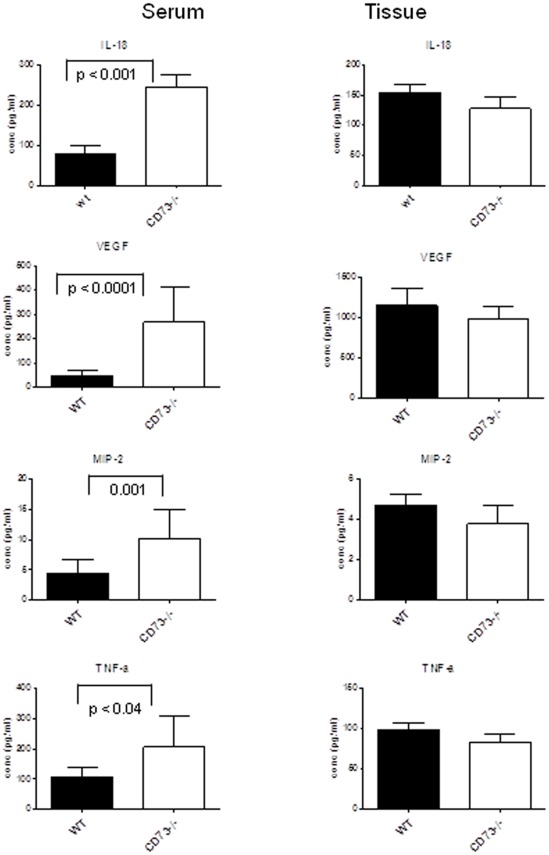
The levels of IL-18, VEGF, MIP-2 and TNFα is increased in the serum but not in the kidney tissue of CD73^−/−^mice. The levels of IL-18, VEGF, MIP-2 and TNFα were analyzed in serum and kidney tissue of CD73^−/−^ and WT mice using BioPlex analysis. 7 animals from each mice group were analyzed. Data are presented as mean l ± SEM. Level of significance as indicated according to according to Mann-Whitney-U-test (p<0.005).

To find the missing link between podocyte damage, kidney injury and the adenosine generating CD73, we analyzed a panel of chemokines, cytokines and angiogenetic factors in kidney tissue and serum of CD73^−/−^ compared to WT mice. The following proteins were quantified in tissue lysates and serum samples: VEGF, fibroblast growth factor (FGF-basic), platelet derived growth factor (PDGF-basic), tumor necrosis factor- α (TNF-α), the interleukines IL-1α, IL-1β, IL-2, IL-6, IL-9, IL-10, IL-12p40, IL-12p70, IL-13, IL-15, IL-17, IL-18, granulocyte macrophage colony stimulating factor (GM-CSF), granulocyte stimulating factor (G-CSF), monocyte stimulating factor (M-CSF), as well as the chemokines CCL2 (MCP-1), CCL5 (RANTES), CXCL2 (MIP-2), CXCL9 (MIG), and the CXCL1 homologue KC. Among these cytokines and chemokines, IL-18 was strongly elevated in serum of CD73^−/−^ mice compared to WT control mice (244.2±30.2 pg/ml; n = 7, vs. 105.5±15.3 pg/ml; n = 7, p<0.005, Figure 8), In parallel to IL-18, VEGF, MIP-2/CXCL-2 and TNF-α were also increased (Figure 8) while PDGF and FGF levels were unaltered (Table 1). Altogether, we saw elevated cytokines and chemokines in the serum compared to the renal tissue and this underlines the inflamed status of the endothelium (Table 1).

**Table 1:** Proinflammatory pattern of angiogenetic factors, chemokines and cytokines observed in the serum but not in the renal tissue of CD73^−/−^mice.

Marker	Serum WT mice	Serum CD73^−/−^mice	Level ofsignificance	Tissue WT mice	Tissue CD73^−/−^ mice	Level of significance
FGF basic	68.43±90.55	120.30±91.63	ns	287.88±41.55	254.04±120.73	ns
PDGF-bb	36796.66±1070.25	57702.83±528	ns	3462.99±851.66	3127.16±472.25	ns
IL1α	16.73±8.37	30.33±24.47	ns	101.35±73.64	63.40±12.85	ns
IL-1β	52.26±41.3	94.30±110.10	ns	197.25±49.8	150.5±74.29	P<0.01
IL-2	11.53±3.28	14.83±7.72	ns	106.78±26.49	91.98±20.79	ns
IL-6	12.25±14.86	24.59±29.37	ns	23.25±5.17	20.46±6.19	ns
IL-9	190.84±34.42	478.89±246.80	ns	1457.10±256.52	1336.33±313.65	ns
IL-10	75.36±41.24	127.40±162.73	ns	154.96±48.5	113.33±51.58	P<0.03
IL12p40	1279.11±575.13	845.44±312.50	ns	70.65±12.55	60.63±20.21	ns
IL12p70	167.03±109.70	471.78±506.15	ns	238.76±82.92	181.98±75.35	ns
IL-13	295.0±88.76	526.76±502.36	ns	526.76±502.36	858.22±460.30	P<0.03
IL-15	150.59±95.78	629.87±577.14	ns	759.34±281.68	610.30±396.29	ns
IL-17	33.02±43.95	116.17±97.55	ns	19.59±3.88	15.37±4.29	P<0.01
IFN- γ	117.337±71.99	434.80±464.21	ns	156.98±33.79	123.31±46.41	P<0.03
GM-CSF	77.67±33.52	171.59±88.95	ns	39.90±8.31	48.58±5.3	P<0.01
G-CSF	236.30±173.08	374.43±264.30	ns	55.20±16.42	68.26±14.24	P<0.02
M-CSF	961.18±175.77	784.99±158.73	ns	12.46±2.50	13.94±10.02	ns
MCP-1	596.45±454.34	214.08±131.66	ns	196.06±24.21	173.21±39.40	P<0.03
Rantes	24.10±17.87	28.81±18.09	ns	32.83±49.94	39.27±21.94	ns
MIG	1049.88±1439.80	1821.43±2001.46	ns	1375.72±327.69	1420.94±934.24	ns
KC	Not detectable	51.09±5.86		63.64±14.08	52.35±23.27	ns

The levels of IL1-β, IL-2, IL-12p40, IL-15, IL-17, IFN-γ, GM-CSF, G-CSF, IL-13, MCP-1, interleukins 3, 4, 5, 10, 12p70, 13, M-CSF, MIG and RANTES were screened in the serum and renal tissue of CD73^−/−^ and WT mice using BioPlex analysis. The serum levels of most measured cytokines and chemokines were slightly but not significantly elevated in the mutants as compared to the wild type controls, suggesting a proinflammatory pattern. The renal tissue levels were unaltered or even reduced in the CD73^−/−^mice compared to WT.

## Discussion

This study explored the functional consequences of a lack of CD73 in the kidney in mice from six weeks to six months. We found renal injury in otherwise unstressed animals, with a pronounced low molecule proteinuria and a reduction of overall renal function in CD73^−/−^ mice ageing 30 weeks. Although the renal physiology and pathophysiology has been investigated in several studies in CD73^−/−^mice [15], [23], [14] the pronounced low molecule proteinuria found in the present study remained undetected so far. All animals previously studied were only 2 months and urinary protein content tended to be higher in one study, without reaching significance [14]. In our study the kidney pathologies were followed up 6 month and revealed that the renal effects are of an autoimmune phenotype and requires time to develop.

Detailed histological analysis revealed tubulointestitial nephritis and glomerulitis. The cellular infiltrates of the renal cortex comprised CD11bGR1positive monocytes and CD8 T cells that are likely to be activated due to expression of the IL-2 receptor α-chain CD25. In contrast, no CD4+T helper cells were detectable suggesting that Treg did not infiltrate into kidney tissue. Remarkably, this infiltration was accompanied by glomerular complement and IgG deposition which indicates the involvement of a humoral response. The nephropathology of the CD73^−/−^mutant therefore clearly is of immunologic origin.

We furthermore observed reduced and swollen podocytes with a lowered expression of the cytoskeletal marker nephrin and synaptopodin as well as reduced endothelial fenestration, swelling of the glomerular endothelium and endotheliosis. Adjacent to the glomerular basement membrane, podocytes and endothelium assemble the glomerular filtration barrier (GFR), and the inflammatory changes of the GFR observed can explain the low molecule proteinuria observed in the present study. The reduced number of podoytes was not associated with apoptosis according to TUNEL analysis, but may relate to a decreased capacity to proliferate. The moderate changes in podocyte phenotyp were not associated with albuminuria in mutant mice being up to 35 week old when compared to control animals.

Earlier studies from our group [14] showed, that under control conditions, tissue levels of adenosine in the WT were 20.9±12.2 nmol g^−1^ kidney weight and were reduced by 76% in the CD73 knockout (p<0.005). However, it must be recognized that total tissue adenosine content consists of an extra- and intracellular fraction comprising also a protein-bound fraction (SAH-hydrolase). Since intercellular adenosine levels remained unchanged, the observed changes relate to a profound decrease of adenosine in the extracellular space [14]. Sites of CD73-derived adenosine production in the kidney are clearly peritubular fibroblasts and mesangial cells. Thus, the observed inflammation and injury most likely relate to these structures of adenosine production within the kidney. We found that lack of the CD73 was associated with cellular infiltrates in the tubulointerstitium and with interstitial fibrosis as judged by the collagen deposition in 30 week old mutant mice, but not with tubular apoptosis. Furthermore, the presence of the CD73 in renal mesangial cells was recently shown to preserve the glomerular filtration barrier via A2A receptor signaling and to stabilize the normal structure of podocyte foot processes, slit diaphragms, and actin cytoskeleton [20]. Consequently our results show that long term lack CD73-derived adenosine causes damage of these structures altering GFR.

As to the mechanisms underlying this autoimmune nephropathology, two different possibilities must be considered.

Firstly, CD73 fulfills an important barrier function, and CD73 deficiency on the endothelium promotes extravasation and migration of PBMCs into the tissue of peripheral organs [23]. This unselectively enhanced endothelial permeability is probably mediated by the A2B receptor [23]. In line with this, CD73^−/−^mice with cardiac grafts showed a decreased graft survival associated to vasculopathy and a loss of the endothelial barrier function accompanied by a reduced expression of the A2b receptor [24]. Endothelial leakiness may be additionally influenced by the enhanced serum levels of VEGF as identified in this study [25].

Secondly, extracellular adenosine is a well-described immune modulator acting via activation of adenosine receptors on various immune cells. The adenosine A2A receptor regulates the T cell mediated responses via T-cell receptor (TCR)-controlled effector functions [26]. In a so-called “2-danger-signal” model, it is suggested that after activation of immune cells with a first cytokine response in the inflamed tissue e g. by certain pathogens, the temporary activation of the CD73 evokes defensive effector functions. This was exemplarily shown within bacterial peritonitis in mice [27] where upregulation of CD73 led to an increase of extracellular adenosine as a second signal, indicating danger from overactive immune responses to prevent excessive collateral damage and destruction of normal tissues [28]. Therefore, lack of CD73 is likely to predispose to an enhanced inflammatory activity of immune cells with subsequent tissue damage. This hypothesis is strengthened by the observed upregulation of the cytokines IL-18, TNFα and the chemoattractant MIP-2 in the serum. Also other cytokines and chemokines such as IL-1α, IL-1β, IL-2, IL-6 IL-12p40, IL-15, IL-17, IFN- α and the colony-stimulating factors GM-CSF, G-CSF as well as the Th-2 cytokine IL-10, IL-13, and the chemokine CCL2 (MCP-1) were found to be increased in serum as well as total IgG. Surprisingly, tissue levels of IL-18, TNFα and MIP-2 remained unaltered, and it is possible that changes of local cellular infiltrates remained undetected given the tissue background of chemokines and cytokines.

The elevated levels of cytokines and the chemokine MIP-2 are presumably derived from the inflamed endothelium and circulating immune cells as well as from extrarenal sources in which adenosinergic feedback inhibition is lacking. This may also relate to compromised T-cell function, since in-vitro studies found that lack of CD73-derived adenosine enhances cytokine and chemokine relase of CD4+T-cells (T effector cells) which is mediated by NF-κB. [29]. In line with this, A2AR stimulation was found responsible for the activity state and cytotoxic effects of T cells in a mouse model of coronary artery clamp and myocardial ischemia [30]. Of particular interest is the observed increase in IL18. An association of IL-18 with renal tubular injury was documented in several models. IL-18 may promote fibrosis as shown in a model of obstructive renal disease through a Fas ligand-dependent mechanism [31]. The elevated levels of IL-18 in the serum of CD73^−/−^mice may be related to the finding that ATP acts as an agonist to promote stimulus-induced secretion of IL-1β and IL-18 in human monocytes [32] and interestingly ATP is known to be released from neutrophils [33], monocytes/macrophages [34], and T cells [35]. IL-18 activates fibroblast and increases the synthesis of extracellular matrix components and fibroblast migration [36]. IL-18 was shown to induce adhesion molecules [36] as documented for VCAM-1 in the vasculature of CD73^−/−^mice [8] and the leukocyte chemoattractant MIP-2 (CXCL-2) as observed in the present study.

In CD73 deficient mice, T and B lymphocyte trafficking into lymphatic tissue and immunological imprinting of these cells is decreased [37]. It is also known that adenosine prevents the priming of naïve CD8 cytotoxic T cells [26] by antigen-presenting cells. In our study, IgG deposition in the glomeruli was associated with the presence of CD25+activated CD8+T cells. Thus, the increased amount of CD8+T cells in renal tissue of CD73^−/−^mice may represent this state of lack of tolerance. Diminished adenosine also turns local macrophages or dendritic cells, e. g. CD 11b/GR1 positive monocytes, into an activated phenotype [38].

In summary the present study demonstrates that chronic lack of CD73 leads to autoimmune interstitial nephritis and glomerulitis with subsequent renal injury and low molecule proteinuria in older-aged CD73^−/−^mice. The underlying mechanisms involve enhanced secretion of IL-18, TNF α, and MIP-2 most likely produced by immune cells lacking adenosinergic feedback. In addition a proinflammatory phenotype of the vasculature might contribute. We expect that the phenotyp already observed in the unstressed mutants becomes aggravated in glomerulonephritis and kidney transplantation. In fact, the use of adenosine receptor agonists has already been shown to be beneficial in a model of lupus nephritis [19] and purinergic therapies might be a promising in the future for nephroprotection in kidney inflammation.
